# Comparative Study of Nanocrystalline Dysprosium Oxide Thin Films Deposited on Quartz Glass and Sapphire Substrates by Means of Electron Beam

**DOI:** 10.3390/nano16010010

**Published:** 2025-12-20

**Authors:** Faisal Alresheedi

**Affiliations:** Department of Physics, College of Science, Qassim University, Buraidah 51452, Saudi Arabia; f.alresheedi@qu.edu.sa

**Keywords:** thin films, dysprosium oxide, optical properties, nanomaterial, anti-reflective

## Abstract

In this study, nanocrystalline dysprosium oxide (Dy_2_O_3_) thin films were deposited on sapphire and quartz glass substrates by an electron beam evaporation technique to comparatively evaluate the influence of substrate type on their structural and optical properties. X-ray diffraction (XRD) confirms that all films exhibit a polycrystalline nature and possess a cubic-type structure. The Debye–Scherrer equation was used to determine the average crystallite size and it was found that the film deposited on quartz glass substrate is slightly larger than the film deposited on the sapphire substrate. Scanning electron microscopy (SEM) revealed a granular morphology for the sapphire film and a more compact, pore-free surface for the quartz film. Spectroscopic ellipsometry (SE) and UV-Vis spectrophotometry were employed to extract the optical constants and reflectance behavior, respectively. The film on sapphire exhibited a lower refractive index, higher extinction coefficient, and reduced reflectance, confirming its enhanced anti-reflective performance. The study provides new insights into how the substrate affects the optical properties of Dy_2_O_3_ thin films. This study demonstrates that sapphire is a more suitable substrate for enhanced anti-reflective and optoelectronic applications.

## 1. Introduction

Rare-earth metal oxides such as dysprosium oxide (Dy_2_O_3_) have attracted considerable attention due to their high thermal and chemical stability, wide band gap, and large dielectric constant [1,2,3,4]. These characteristics make Dy_2_O_3_ an appealing candidate for optoelectronic and protective coating applications. Dy_2_O_3_ possesses a high refractive index, making it suitable for use in optical devices such as modulators, anti-reflection coatings, optical filters, and switches [5,6,7,8]. Moreover, its excellent thermodynamic stability enables it to serve as a protective layer against corrosion in stainless steel and optical elements operating under high temperatures [9,10,11]. In microelectronics, Dy_2_O_3_ is employed as a high-k dielectric material and in resistive switching devices, owing to its low leakage current and strong insulating behavior [12,13,14]. Various techniques have been used to deposit Dy_2_O_3_ thin films, including reactive magnetron sputtering, thermal evaporation, electron beam evaporation, and atomic layer deposition [15,16,17,18]. Each deposition method significantly influences the film’s morphology, crystallinity, and optical constants. For instance, sputtered Dy_2_O_3_ films exhibit high density but increased surface roughness, while electron beam-evaporated films provide smoother and more uniform surfaces at relatively low substrate temperatures [19,20].

Several studies have reported optical properties of Dy_2_O_3_ films, including absorption coefficient (α), reflectance (R), band gap (Eg), and refractive index (n) [21,22,23]. Goswami and Varma [21] found a refractive index of 1.85 for bulk Dy_2_O_3_, while other studies reported band gap values ranging between 4.26 and 4.8 eV [21,24]. Cherif et al. [25] demonstrated that the electrical behavior of Dy_2_O_3_ on silicon is temperature-dependent, while Ramay et al. [26] improved the anti-reflective performance of Dy_2_O_3_ films deposited on silicon using magnetron sputtering. However, despite extensive studies on deal Dy_2_O_3_ thin films, comparative investigations of substrate influence remain limited, especially when it comes to the effects of quartz glass and sapphire substrates on the structural order, optical constants, and anti-reflective behavior of the film. Quartz glass is an amorphous substrate with relatively low surface energy, while Sapphire (Al_2_O_3_) is crystalline with a high lattice order and superior thermal conductivity. These differences are expected to significantly influence film nucleation, crystallite growth, and optical properties, yet systemic comparison under identical deposition conditions are scarce [27]. Understanding these substrate-induced effects is crucial for optimizing Dy_2_O_3_ thin films for optoelectronic and semiconductor applications, where control refractive index and surface morphology is required.

Although substrate effects on rare-earth oxide thin films have been reported previously [28,29,30,31], most studies focus on thicker films, single property analysis, or different deposition conditions, making it difficult to isolate substrate-driven mechanisms. The key new insight of the present work lies in demonstrating that, in the ultrathin regime (~13 nm), the optical response of Dy_2_O_3_ is governed primarily by substrate-induced strain and surface morphology rather than crystallite size alone. By deliberately selecting amorphous quartz glass and crystalline sapphire (Al_2_O_3_) as model substrates—representing two extreme cases of surface order, thermal conductivity, and lattice matching—this study provides a controlled platform to decouple the roles of lattice mismatch, defect density, and surface porosity on the refractive index, extinction coefficient, and reflectance. Unlike previous reports, the present work establishes a direct structure–optical correlation using a combined XRD–SEM–spectroscopic ellipsometry–UV–Vis approach under identical deposition and annealing conditions. Therefore, the novelty of this study is not the introduction of a new deposition method, but the mechanistic clarification of how substrate nature controls anti-reflective behavior in ultrathin Dy_2_O_3_ films, which is highly relevant for optoelectronic coatings where thickness, absorption losses, and interface effects are critical.

In this study, nanocrystalline Dy_2_O_3_ thin films were deposited on quartz glass and sapphire substrates using an electron beam evaporation technique. The structural and optical properties these films were analyzed using X-ray diffraction (XRD), spectroscopic ellipsometry (SE), scanning electron microscope (SEM), and UV-Vis spectrophotometry. The optical constants including the refractive index (n) and the extinction coefficient (k) were determined to clarify how the substrate nature effects the film’s microstructure and optical performance. This comparative approach provides new insights into the substrate-dependent behavior of Dy_2_O_3_ thin films and supports their potential use in advanced optical and electronics devices.

## 2. Materials and Methods

**Synthesis.** Dy_2_O_3_ thin films were deposited on sapphire and quartz glass substrates using an electron beam evaporation technique. The deposition was carried out in an ultra-high vacuum system with a base pressure of ~1 × 10^−7^ Torr, while the working pressure during evaporation was typically in the 10^−6^–10^−5^ Torr range, which is consistent with standard e-beam PVD conditions. The process for fabricating both thin films was carried out under the same conditions. This process was carried out at a temperature of 275 °C and a growth rate of 0.2 Å/s. The thin films were subsequently heat-treated at 350 °C for 15 min. The thickness of Dy_2_O_3_ thin films on both substrates was approximately 13 nm, as determined using a quartz crystal rate/thickness monitor equipped with a deposition rate controller.

**Characterizations**: XRD was used to examine the structural characteristics of Dy_2_O_3_ thin films. This technique equipped with Cu Kα radiation (*λ* = 0.0154 nm) and was carried out at room temperature. The measurements were run in the range of 2θ between 10° ≤ 2θ ≤ 90°, at a step scan size of 0.020°. A field emission scanning electron microscope (FE-SEM) (JEOL 7600F) working at 5 kV was used to investigate the morphology of the Dy_2_O_3_ thin films. An M-2000 Ellipsometer instrument was used to examine the optical properties (J.A. Woollam Co., Lincoln, NE, USA) across a wavelength range of 300 to 1800 nm. The measurements were taken at room temperature and an incident angle of 70°, and Cauchy’s model was used to perform data fitting. The model function was fitted to the collected data using the WVASE32 software developed by J.A. Woollam Company to obtain the values of the optical constants (refractive index and extinction coefficient). The reflectance of Dy_2_O_3_ thin films was also examined at the normal incident using a double-beam UV–Vis spectrophotometer (JASCO UV–Vis–NIR 670). These measurements were obtained across a wavelength range of 300 to 700 nm at room temperature.

## 3. Results and Discussions

### 3.1. Structure Analysis of Dy_2_O_3_ Thin Films

**Figure 1:**
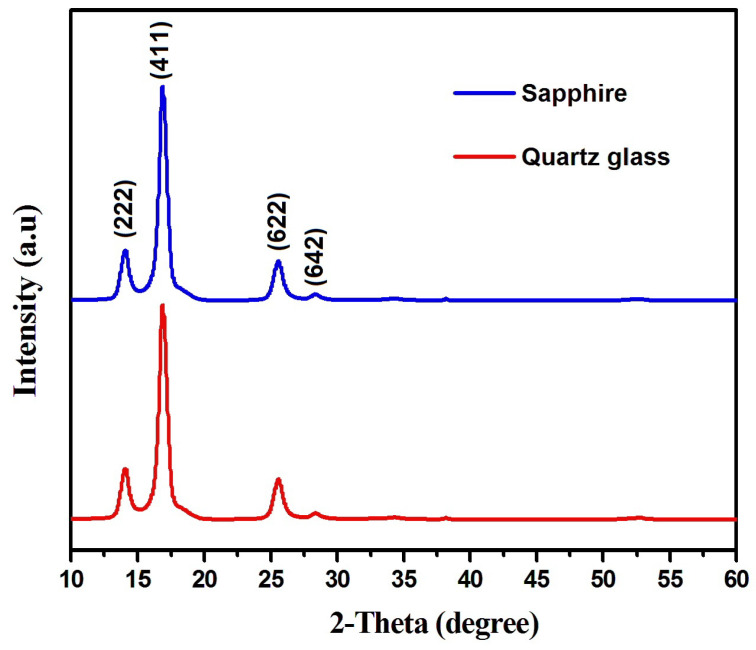
X-ray results for Dy_2_O_3_ thin films deposited on the quartz glass and sapphire substrates.

XRD analysis was performed to investigate the structural. nature of Dy_2_O_3_ thin layers deposited on quartz glass and sapphire substrates. The data from the International Centre for Diffraction Data (ICDD) was used to determine the peaks [32] (PDF 00-002-0900 (Dy), PDF 01-086-0127 (Dy_2_O_3_), PDF 00-019-0430 (Dy(OH)_3_), PDF 01-086-2229 (Dy hydroxide carbonates)). It can be seen in Figure 1 that there is a polycrystalline structure and preferential orientation of the deposited thin films in the (4 1 1) direction. It is evident from the patterns of the deposited thin films hat the structure of the oxide thin films is of a cubic crystalline nature and conforms to the ICDD pattern # 00-009-0197. Significantly higher values of the full width at half maximum (FWHM) and intensity are implied by the broadness of the pronounced (4 1 1) diffraction peak, noted at 2θ = 16.79°, 2θ = 16.58° for the quartz glass and sapphire thin films, respectively. The average crystallite size (D) was calculated using the Debye–Scherrer equation given below [33]:

(1) D=0.9 λΔ2θ cosθ

In this equation, λ refers to the wavelength of the X-rays (λ = 0.15406 nm),0.9 refers to the Scherrer factor, (θ) denotes the position of the diffraction peaks, and ∆(2θ) is the full width at half maximum of the peak (4 1 1) and has values of 0.584 and 0.622 for the quartz glass and sapphire substrates, respectively. The average crystallite size (D) of the Dy_2_O_3_ thin films was calculated using the Debye–Scherrer equation. These values were 13.75 nm and 12.91 nm for the quartz glass and sapphire substrates, respectively. The larger crystallite size on quartz glass can be attributed to its amorphous nature, which provides fewer well-defined nucleation sites, leading to slower initial nucleation and allowing larger grains to grow during formation [34,35]. In contrast, the crystalline sapphire substrate has a higher density of nucleation sites due to its ordered surface structure and lattice mismatch with Dy_2_O_3_ resulting in increased interfacial strain and the formation of smaller crystallite [33,34]. Moreover, sapphire’s higher thermal conductivity enhances heat dissipation during deposition, which reduces adatom mobility and further limits crystallite growth. The equation given below was also used to determine the dislocation density (δ) for the thin films:

(2) $$\delta =\frac{1}{D^{2}}$$

Table 1 presents the computed values of the dislocation density, with nano-scale crystallinity exhibited by both thin films. It can be noted that the δ value of the Dy_2_O_3_ thin film grown on the quartz glass substrate is slightly less than the films deposited on the sapphire substrate. The obtained results suggest that the Dy_2_O_3_ thin film deposited on the quartz substrate has improved crystallinity than the film deposited on the sapphire substrate.

**Figure 2:**
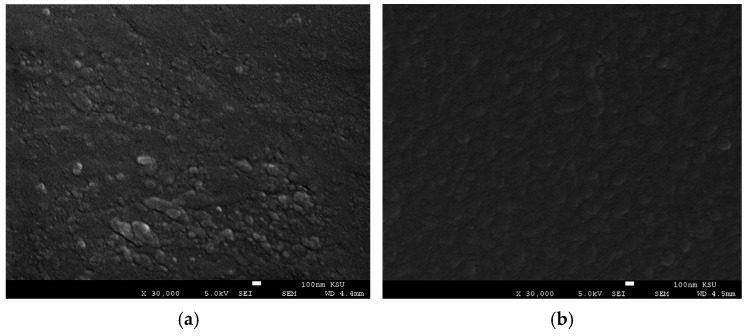
(**a**). SEM image of Dy_2_O_3_ thin film deposited on the sapphire substrate. (**b**). SEM image of Dy_2_O_3_ thin film deposited on the quartz glass substrate.

Figure 2a displays the thin film deposited on the sapphire substrate, which exhibits a granular surface composed of interconnected nanocrystallites. The particles appear relatively large, agglomerated, and irregular in shape, with noticeable cluster formation. In contrast, the film deposited on the quartz glass substrate (Figure 2b) also consists of surface nanocrystallites but forms a denser and more compact layer with minimal surface voids. Additionally, this film demonstrates a relatively uniform, fine, and smooth particle distribution, with grains that are mostly spherical or slightly irregular in shape. The estimated average grain sizes indicate that the Dy_2_O_3_ thin film deposited on the sapphire substrate has an average grain size of approximately 50 nm, whereas the thin film grown on quartz glass exhibits a smaller average grain size of about 30 nm. Although the crystallite size of Dy_2_O_3_ thin films deposited on both substrates was nearly identical (nearly 13 nm), the SEM analysis revealed larger surface grains for the thin film grown on sapphire (50 nm) compared to quartz glass (30 nm). This difference can be attributed to the higher surface energy and crystalline nature of the sapphire substrate, which enhances adatom mobility during growth. On the other hand, the amorphous quartz substrate restricts surface diffusion, causing smaller and more compact grains [34,35]. The crystallite size estimated from the Scherrer equation (~13 nm) represents the size of coherently scattering domains within the Dy_2_O_3_ thin film. In contrast, the SEM images reveal much larger grains (30–50 nm), which correspond to surface morphological units composed of multiple crystallites. Such a discrepancy is typical for polycrystalline oxide thin films, where each SEM-visible grain may contain several coherently diffracting domains separated by low-angle boundaries or defect-rich regions. Furthermore, the Scherrer-derived crystallite size should be considered a lower-bound estimate, since peak broadening may also include contributions from microstrain, lattice defects, and anisotropic broadening. No advanced deconvolution beyond the instrument broadening correction was applied. This microstructural hierarchy explains the difference between the XRD-derived crystallite size and the larger grain size observed in SEM.

### 3.2. Optical Properties

The alterations in polarization resulting from the interaction of light with the sample structure were examined using spectroscopic ellipsometry (SE). Ellipsometry allows the determination of optical constants, film thickness, and various physical features of thin films [35,36,37,38,39]. Measurements were performed at room temperature with the detector set at an angle of 70° to acquire the SE data. The mean square error (MSE) of the instrument was recorded prior to analysis to ensure data reliability. The SE data were modeled using a multilayer structure consisting of substrate/Dy_2_O_3_ film/effective medium approximation (EMA) surface layer/ambient. The EMA layer, composed of a 50:50 mixture of Dy_2_O_3_ and voids, was introduced to account for surface porosity, density fluctuations, and incomplete film densification, which are common in ultrathin electron-beam-evaporated oxide films. It is important to note that the EMA layer thickness does not correspond to physical surface roughness; for ultrathin films (~13 nm), it represents an effective optical approximation capturing microstructural heterogeneity such as island coalescence and sub-nanometer porosity. Consequently, the extracted EMA layer thickness (~12–13 nm) should not be interpreted as topographical roughness, but rather as an indicator of transitional ultrathin film growth. This interpretation is consistent with SEM observations, which show granular but continuous film coverage without large-scale height variations.

**Figure 3:**
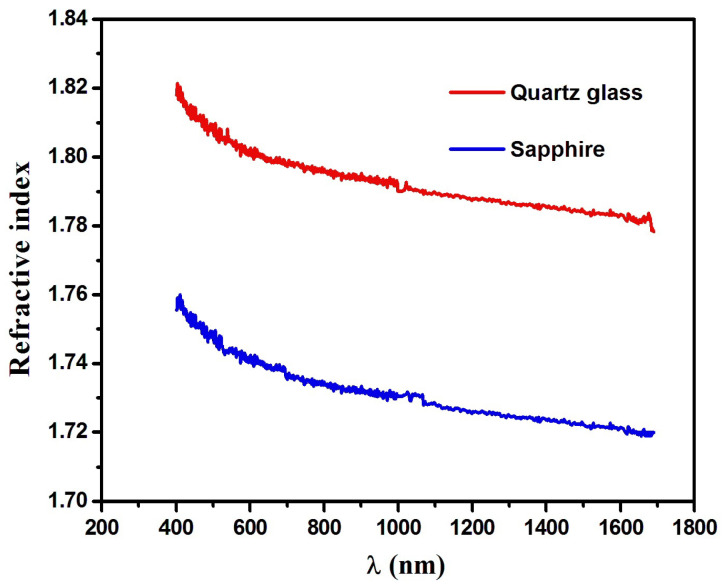
Refractive index of Dy_2_O_3_ thin films grown on the quartz glass and sapphire substrates.

**Figure 4:**
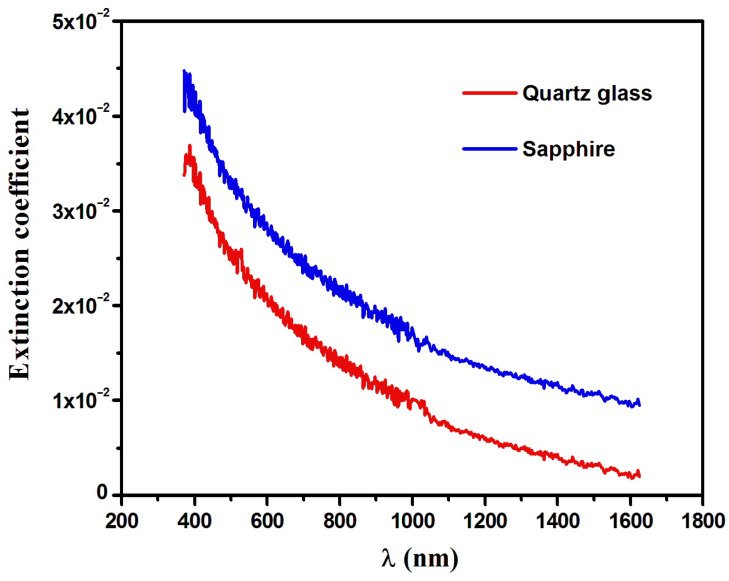
Extinction coefficients of Dy_2_O_3_ thin films grown on the quartz glass and sapphire substrates.

The film thickness of Dy_2_O_3_ on both quartz glass and sapphire substrates was determined to be approximately 13 nm, in agreement with XRD measurements. To extract the optical constants in the low-absorption region, the Cauchy model was applied, allowing accurate determination of the refractive index (n) and film thickness from the ellipsometry parameters Psi (ψ) and Delta (Δ). The quality of the ellipsometric fits was verified by low MSE values, indicating good agreement between experimental and modeled data. The optical constants—refractive index (n) and extinction coefficient (*k*)—were stable across multiple fitting iterations, confirming the robustness of the model despite the ultrathin nature of the films. The refractive index (n) of the films is shown in Figure 3. It decreases with increasing wavelength, and at 632.8 nm, n values are 1.80 and 1.73 for quartz glass and sapphire substrates, respectively. These values are consistent with prior studies [26], where Dy_2_O_3_ films deposited on silicon exhibited a refractive index of 1.71. The **extinction coefficient (*****k*****)**, plotted in Figure 4, decreases with increasing wavelength. The Dy_2_O_3_ films on sapphire and quartz glass show *k* values of 26.4 × 10^−3^ and 18.9 × 10^−3^, respectively, indicating higher optical absorption for films on sapphire. This enhanced absorption is attributed to substrate-induced strain, interfacial defects, and surface porosity caused by lattice and thermal mismatch between Dy_2_O_3_ and sapphire, which promote the formation of localized energy states. In contrast, films on quartz, due to the amorphous nature of the substrate, exhibit lower extinction coefficients and slightly higher refractive indices, reflecting a more uniform microstructure with fewer defects [10,30,31,40,41,42].

The obtained *k* values were used to calculate the absorption coefficient (α) of both Dy_2_O_3_ of thin films by using the following equation:

(3) $$\alpha =\frac{4\pi k}{\lambda }\;$$

Here, k refers to the extinction coefficient and λ represents the wavelength. The absorption coefficient (α) spectra is examined to obtain vital information regarding the optical band gap structure and the interband shifts in the material. The relationship given below describes the absorption coefficient for a transition permitted directly,

(4) $$\left(\alpha E\right){\propto \left(E-E^{*}\right)}^{1/2}$$

**Figure 5:**
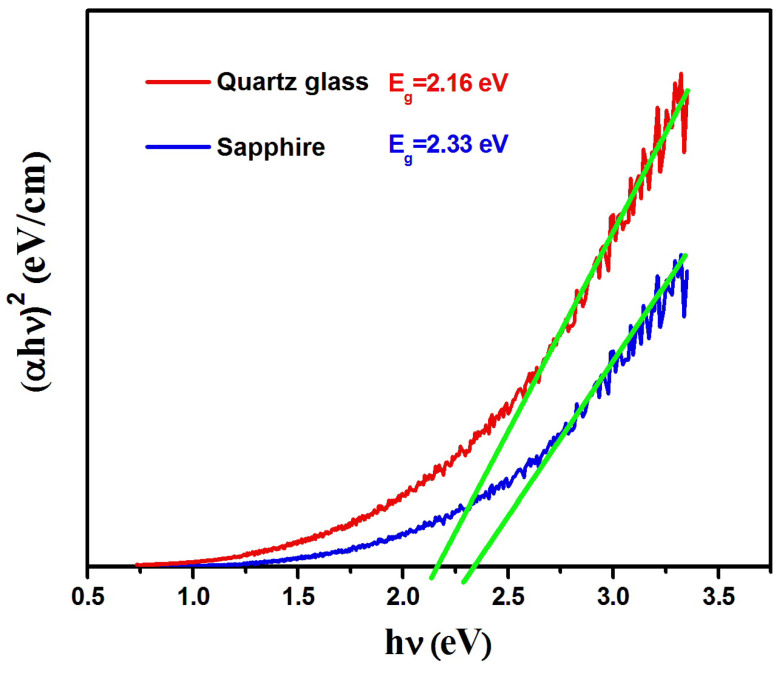
${\left(\alpha hv\right)}^{2}$ vs. photon energy spectra (hv) for Dy_2_O_3_ thin films grown on the quartz glass and sapphire substrates.

Here, (E) refers to the photon energy (E = hv) and (E*) denotes the energy of the allowed optical transition. The plot of (αE)^2^ against photon energy (E) for both thin films can be seen in Figure 5. The optical band gap value was obtained at the intercept of the linear fit with the energy axis at (αE)^2^ = 0. This plot was used to find the value of the energy of the allowed optical transition of Dy_2_O_3_ thin films and was found to be 2.16 and 2.33 eV for the thin film deposited on quartz glass and sapphire substrates, respectively. These energy values, being lower than the Dy_2_O_3_ band energy, are attributed to the allowed ^4^F_9/2_-^6^H_15/2_ transition of Dy^3+^ ion [43,44,45].

### 3.3. Reflectance Study

**Figure 6:**
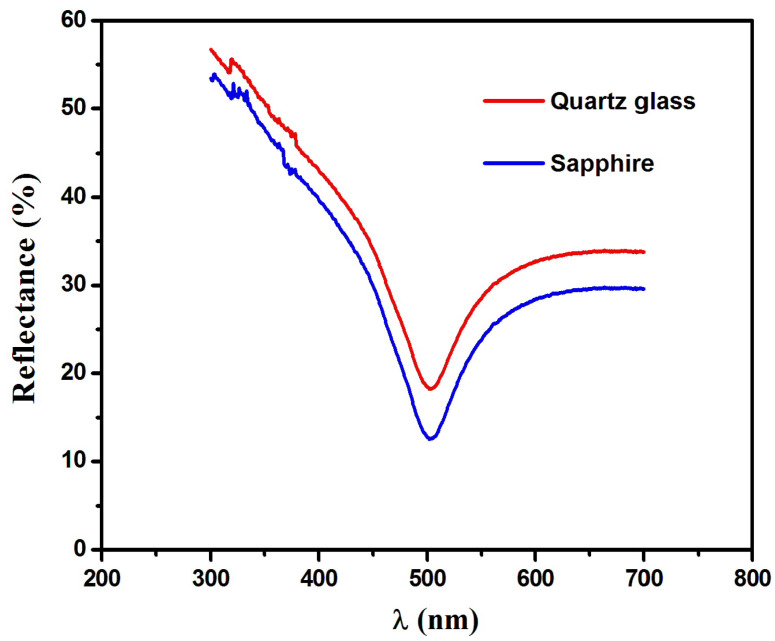
Reflectance spectra as a function of wavelength for Dy_2_O_3_ thin films grown on the quartz glass and sapphire substrates.

As shown in Figure 6, the percentage reflectance of the oxide thin films was determined using UV–Vis spectrophotometry. The reflectance spectra exhibit a pronounced decrease in reflectance with increasing wavelength up to approximately 500 nm, beyond which the reflectance gradually increases. For the Dy_2_O_3_ thin film deposited on the sapphire substrate, approximately 13% of the incident radiation is reflected, whereas the thin film grown on quartz glass reflects about 18% of the visible light. This indicates a significantly higher light absorption for the sapphire-supported thin film. The enhanced absorption and reduced reflectance observed for the sapphire substrate arise from a combination of substrate-induced interfacial strain, defect density, and surface morphology. XRD analysis reveals a slightly higher dislocation density for the film deposited on sapphire, which can be attributed to lattice mismatch between Dy_2_O_3_ and the crystalline sapphire substrate. These mismatch-induced defects generate localized energy states within the bandgap, leading to enhanced sub-bandgap absorption and a corresponding increase in the extinction coefficient (*k*). The increased optical losses associated with these defect states contribute directly to the reduced reflectance observed for the sapphire-supported thin film. In addition to interfacial defects, surface morphology plays a crucial role. SEM images (Figure 2a) show the presence of pores and surface irregularities in the thin film deposited on sapphire. These morphological features enhance light scattering and absorption at the film surface, effectively reducing the refractive index (*n*) and further suppressing reflectance. Therefore, the enhanced sub-bandgap absorption and reduced refractive index on sapphire originate from the combined effects of lattice-mismatch-induced defects and surface porosity, rather than from grain size alone. In contrast, the Dy_2_O_3_ thin film grown on amorphous quartz glass experiences minimal interfacial strain, resulting in a lower defect density and a more uniform, dense microstructure. Consequently, this thin film exhibits a lower extinction coefficient, a higher refractive index, and higher reflectance, consistent with reduced optical absorption and scattering losses. The reflectance minimum (corresponding to the absorbance maximum) observed at 500 nm (2.33 eV) is lower than the reported optical bandgap of Dy_2_O_3_ but closely matches the optical transition energy extracted from spectroscopic ellipsometry via the extinction coefficient (*k*). This feature is attributed to the allowed electronic transition of Dy^3+^ ions (^4F_9_/_2_ → ^6H_15_/_2_). Despite the slightly larger crystallite size of the sapphire-supported thin film (13.75 nm) compared to that on quartz glass (12.91 nm), the optical response is dominated by substrate-induced effects rather than crystallite size. To frame our results, the substrate-dependent optical behavior of other oxide thin films has been discussed in previous studies [28,29]. These works highlighted that lattice mismatch, defect density, and surface morphology significantly influence the optical constants and anti-reflective performance of rare-earth oxide thin films. In the present study, ultrathin (~13 nm) Dy_2_O_3_ films were deposited on amorphous quartz glass and crystalline sapphire under identical conditions. By combining X-ray diffraction (XRD), scanning electron microscopy (SEM), spectroscopic ellipsometry (SE), and UV–Vis spectrophotometry, we directly correlate substrate-induced structural variations with optical properties such as refractive index, extinction coefficient, and reflectance. This approach allows a mechanistic understanding of how lattice mismatch, defects, and surface morphology jointly affect the optical performance, which was not simultaneously addressed in the cited studies [28,29].

## 4. Conclusions

Dy_2_O_3_ thin films were successfully deposited on quartz glass and sapphire substrates via electron-beam evaporation. XRD confirmed that both thin films are polycrystalline with a cubic structure and preferential (4 1 1) orientation, with average crystallite sizes of 13.75 nm (sapphire substrate) and 12.91 nm (quartz substrate). Optical characterization using spectroscopic ellipsometry and UV–Vis spectrophotometry revealed that the refractive index (n) decreases and the extinction coefficient (*k*) increases for sapphire-supported films compared to quartz. The enhanced absorption and reduced refractive index on sapphire arise from a combination of interfacial strain, higher defect density, and surface porosity, as evidenced by XRD and SEM analyses. The absorption spectra show electronic transitions corresponding to the ^4^F_9_/_2_ → ^6^H_15_/_2_ Dy^3+^ energy levels. The reflectance minimum (~13%) observed for Dy_2_O_3_ deposited on sapphire substrate demonstrates improved anti-reflective behavior compared to the thin film grown on quartz substrate (~18%), in agreement with other studies reporting substrate-dependent reflectance for Dy_2_O_3_ and related oxide thin films such as ZrO_2_ and Y_2_O_3_. These comparisons confirm that substrate choice strongly influences optical performance, particularly in the ultrathin film regime. The Cauchy model was applied only in the low-absorption region to extract refractive index and thickness, while *k* in the absorbing region was determined directly from ellipsometry, ensuring reliable absorption coefficients and optical band-gap estimates. The combined effects of substrate-induced strain, defects, and surface morphology dominate the optical response of Dy_2_O_3_ thin films, highlighting the importance of substrate selection for optimizing optical performance in ultrathin oxide layers for optoelectronic and semiconductor devices.

## Figures and Tables

**Table 1:** Summarized results of the calculated crystallite size, dislocation density, refractive index, extinction coefficient, reflectance, and optical band gap for Dy_2_O_3_ thin films deposited on quartz glass and sapphire substrates.

Property	Quartz Glass	Sapphire
Crystallite size (nm)	13.75 ± 0.02	12.91 ± 0.02
Dislocation density (nm^−2^)	5.2 × 10^−3^	5.9 × 10^−3^
Refractive index @ 632.8 nm	1.80 ± 0.01	1.73 ± 0.01
Extinction Coefficient @ 632.8 nm	18.9 × 10^−3^ ± 0.002	26.4 × 10^−3^ ± 0.002
Reflectance % at 500 nm	18 ± 1%	13 ± 1%
Energy (eV)	2.16 ± 0.02	2.33 ± 0.02

## Data Availability

Data are contained within the article.
